# *Sinorhizobium meliloti* integration host factor (IHF) genes affect symbiotic performance of alfalfa ( *Medicago sativa* L.) Symbiotic functions of rhizobial *ihf* genes

**DOI:** 10.3724/abbs.2022110

**Published:** 2022-08-17

**Authors:** Ningning Li, Lanya Zhang, Wenjia Zheng, Dandan Shan, Yawen Wang, Ru-Jie Li, Liangliang Yu, Christian Staehelin, Li Luo

**Affiliations:** 1 Shanghai Key Laboratory of Bio-energy Crops School of Life Sciences Shanghai University Shanghai 200444 China; 2 State Key Laboratory of Biocontrol and Guangdong Key Laboratory of Plant Resources School of Life Sciences Sun Yat-sen University Guangzhou 510275 China

Integration host factor (IHF) is a histone-like bacterial priotein that can sequence-specifically bind to DNA [1]. IHF has diverse effects on gene expression, though these effects are usually not very strong. IHF is required for transcriptional activation of some δ ^54^ promoters in a number of Gram-negative bacteria by assisting transcription factors in forming a transcription-competent open promoter complex [2]. In nitrogen-fixing bacteria, transcriptional activation of nitrogen fixation operons ( *nif* and *fix* genes) usually requires δ ^54^ (an alternative δ factor) and the activator protein NifA [3]. For example, NifA-mediated transcriptional activation of the *nifH* promoter is stimulated by IHF in *Klebsiella pneumoniae*. Similarly, NifA-mediated transcriptional activation of the *nifH* and *nifD* promoters is strongly enhanced by IHF in *Bradyrhizobium japonicum*, whereas only a weak activation of the *nifH* promoter was reported for *Sinorhizobium* ( *Ensifer*) *meliloti* [4]. It was suggested that IHF improves the efficiency and specificity of symbiotic expression of the *fixABCX* operon in rhizobia [5]. IHF can also stimulate the expression of genes of the rhizobial dicarboxylic acid transport (Dct) system. DctD (the response regulator)-mediated activation of the *dctA* promoter was reported for *Rhizobium leguminosarum*, whereas activation of *dctA* in *S*. *meliloti* was found to be independent of IHF *in vitro* [6].

*S*. *meliloti* infects legume host plants such as *Medicago sativa* L. (alfalfa) and *M*. *truncatula* Gaertn. to induce the formation of nitrogen-fixing root nodules. At the early stage of symbiosis, flavonoids such as luteolin secreted by host roots bind to the LysR family regulator protein NodD1. The flavonoid-NodD1 complex subsequently activates the expression of nodulation genes (such as *nodABC*) to synthesize bacterial nodulation signals known as Nod factors [7]. *Medicago* plants perceive Nod factors of *S*. *meliloti* by specific LysM-type receptor kinases to stimulate the expression of genes required for bacterial infection and nodule primordium development [8]. In formed nodules, bacteria are released from infection threads into host cells and differentiate into nitrogen-fixing bacteroids. Expression of the nitrogenase genes *nifHDK* and *fixABC* depends on NifA and also partially on IHF proteins encoded by *ihfA* and *ihfB* [ 5, 6] . Whether *S*. *meliloti* IHF affects alfalfa nodulation at early symbiotic stages has not been investigated, however. In the present work, the symbiotic functions of the *S*. *meliloti infA*/ *B* genes were analyzed in nodulation tests with constructed deletion mutants. Strains and plasmids are shown in Supplementary Table S1 and primers are shown in Supplementary Table S2.

Three *infA*/ *B* deletion mutants of *S*. *meliloti* Sm1021 (Δ *ihfA*, Δ *ihfB* and the double mutant Δ *ihfAB*) were constructed using a suicide plasmid-homologous recombination strategy ( Supplementary Figure S1A). The mutants with correct double-cross recombination event were identified by PCR ( Supplementary Figure S1B,C), and the mutations were confirmed by DNA sequencing. Total RNA was extracted from free-living *Sinorhizobium* cells and reverse transcription PCR (RT-PCR) was performed to determine the transcript levels of *infA*/ *B*. The *infA*/ *B* transcripts could not be detected in the deletion mutants while considerable expression was found in the parent strain Sm1021 ( Supplementary Figure S1D,E). These results confirmed that the *infA*/ *B* deletion mutants were successfully constructed.

Growth curves with LB/MC broth were determined to evaluate the effects of *infA*/ *B* on bacterial growth and proliferation. The results indicated that the logarithmic growth phase was delayed in the three mutants as compared to Sm1021 ( Figure 1A). These results suggest that *ihfA* and *ihfB* can affect growth of free-living *S*. *meliloti* bacteria under the tested condition.

**Figure 1:**
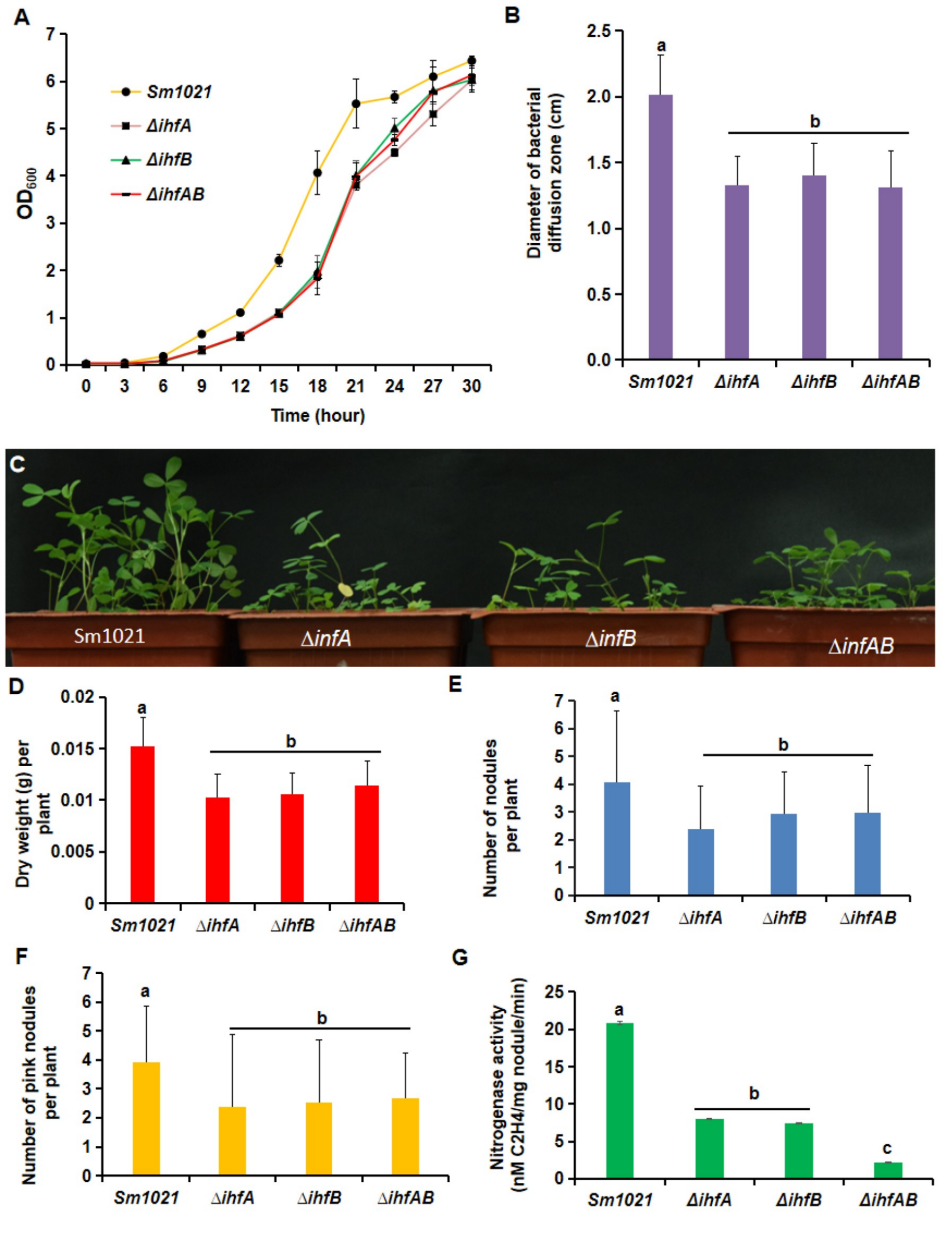
Figure 1 Defects of *ihfA*/ *B* deletion mutants during free-living and symbiotic stages (A) Growth curves of the constructed S. meliloti mutants and the parent strain Sm1021. At time zero, rhizobial cells were diluted with LB/MC broth to an OD 600 of 0.01. The growth of Sm1021, ΔihfA, ΔihfB and ΔihfAB was monitored by measuring the optical density at 600 nm. The data points represent the mean of triplicate samples and the error bars indicate the standard error. (B) Diffusion zone of bacterial cells grown on soft/swimming agar plates. Data are shown as the mean±SD of triplicate samples. (C–G) Results of nodulation tests with indicated strains. Alfalfa plants (10 plants per pot; 3 pots per strain) were harvested four weeks after inoculation. Data are shown as the mean±SD ( n=30). The experiments were repeated four times and a representative experiment is shown. (C) Growth phenotype of test plants at the time of harvest. (D) Shoot biomass (dry weight) of harvested plants. (E) Number of pink nodules per plant. (F) Number of total nodules per plant. (G) Nitrogenase activity of alfalfa nodules as assessed by the C 2H 2 reduction method. Data are shown as the mean±SD from nodules of 30 plants. Different letters indicate significant differences ( P<0.05) as analyzed by one-way ANOVA followed by Tukey’s post hoc test.

Furthermore, cell motility assays using LB/MC swimming plates were performed with the constructed mutants. The results indicated that the three mutants swam obviously slower than Sm1021 ( Figure 1B and Supplementary Figure S2). These observations suggest that there is a genetic connection between *ihfA*/ *B* genes and genes cluster involving in bacterial flagellum biosynthesis and motility.

To evaluate the symbiotic phenotypes of the *ihfA*/ *B* deletion mutants, *S*. *meliloti* suspensions were inoculated onto roots of alfalfa seedlings. Plants were harvested four weeks later. Plants inoculated with the deletion mutants showed reduced growth when compared to those inoculated with Sm1021 ( Figure 1C). The shoot biomass (dry weight) of alfalfa plants inoculated with *ihfA*/ *B* deletion mutants was approximately one third lower than that of plants inoculated with Sm1021 ( Figure 1D). As compared to parent strain Sm1021, the inoculation of three mutants resulted in the impaired nodule formation of host plant. Plants inoculated with the mutants formed fewer nodules both in total number and in pink ( Figure 1E,F). In general, pink nodule is thought of being normal in production of leghemoglobin that is essentially required for the nitrogen fixation process. Furthermore, we measured the nitrogen fixation capacity of the nodules by the acetylene reduction method. The results showed that the nitrogenase activity of nodules induced by the mutants was significantly decreased relative to that of Sm1021 (8.7, 7.3 and 2.1 nmol C _2_H _4_/mg nodule protein/min for Δ *ihfA*, Δ *ihfB* and Δ *ihfAB*, respectively; 20.8 nmol C _2_H _4_/mg nodule protein/min for Sm1021) ( Figure 1G). Taken together, these data indicate that the *ihfA*/ *B* genes are important for nitrogen fixation in alfalfa nodules.

To evaluate the effects of *ihfA*/ *B* deletions on the bacterial nodulation competitiveness, Sm1021 and *ΔihfAB* bacteria were mixed at a 1:1 or 1:9 (Sm1021: *ΔihfAB*) ratio and then inoculated onto alfalfa seedlings. Nodule occupation ratios were determined three weeks after inoculation. The results showed that all of the examined nodules contained Sm1021 at the 1:1 inoculation ratio and that 99% of the nodules were occupied by Sm1021 bacteria at the 1:9 inoculation ratio. These findings show that the *ihfA* and *ihfB* genes promote the establishment of symbiosis in competitive nodulation experiments.

Since IHF interacts with RNA polymerase and transcription factors to regulate transcription, it can be expected that the observed symbiotic phenotype of the *ihfA*/ *B* deletion mutants are resulted from differential gene expression. To test this possibility, total RNA from free-living *S*. *meliloti* cells in LB/MC cultures was extracted for whole genome RNA-seq analysis. As shown in Supplementary Table S3 (q value <0.05 and FC2), 1026, 476 and 1036 genes were found to be differentially expressed in *ΔihfA*, *ΔihfB* and *ΔihfAB* relative to the parent strain Sm1021. The number of significantly upregulated genes was 336, 117 and 206, whereas that of significantly downregulation genes was 690, 359 and 830 respectively in these mutants. Remarkably, the three mutants showed downregulation of chemotaxis and flagellum biosynthesis genes to a varying degree ( Figure 2A). Downregulation of five selected genes (possibly involved in nodulation competitiveness) was verified by quantitative RT-PCR ( Figure 2B). Furthermore, the RNA-seq data indicated a downregulation trend of nodulation genes in the mutants ( Figure 2C). Quantitative RT-PCR confirmed the downregulation of key nodulation genes in the *ΔihfAB* mutant. Expressions of *nodC* and *nodD1* in the mutant were also lower than those in Sm1021 when cells were treated with a flavonoid-containing extract from alfalfa seeds ( Figure 2D). These results indicate that *ihf* genes of *S*. *meliloti* are important for transcriptional activation of key nodulation genes, suggesting that they affect the synthesis of Nod factors. To evaluate the activity of Nod factors produced by the mutants, quantitative RT-PCR was used to determine the transcript levels of the early nodulin gene *MtENOD11* of *M*. *truncatula*, whose expression is known to be rapidly induced by Nod factors [9]. The results showed that the cell suspensions of the *ihfA*/ *B* mutants inefficiently induced the expression of *MtENOD11* in *M*. *truncatula* seedlings when compared to the suspension of Sm1021 or a treatment with purified NodSm-IV(C16:2, S), a tetrameric Nod factor of *S*. *meliloti* ( Figure 2E). These results indicate that *ihfA*/ *B* are regulator genes of Nod factor synthesis. Thus, impaired nodule formation of alfalfa inoculated with *ihfA*/ *B* mutants ( Figure 1D,E) could be due to the downregulation of nodulation genes and reduced Nod factor production.

**Figure 2:**
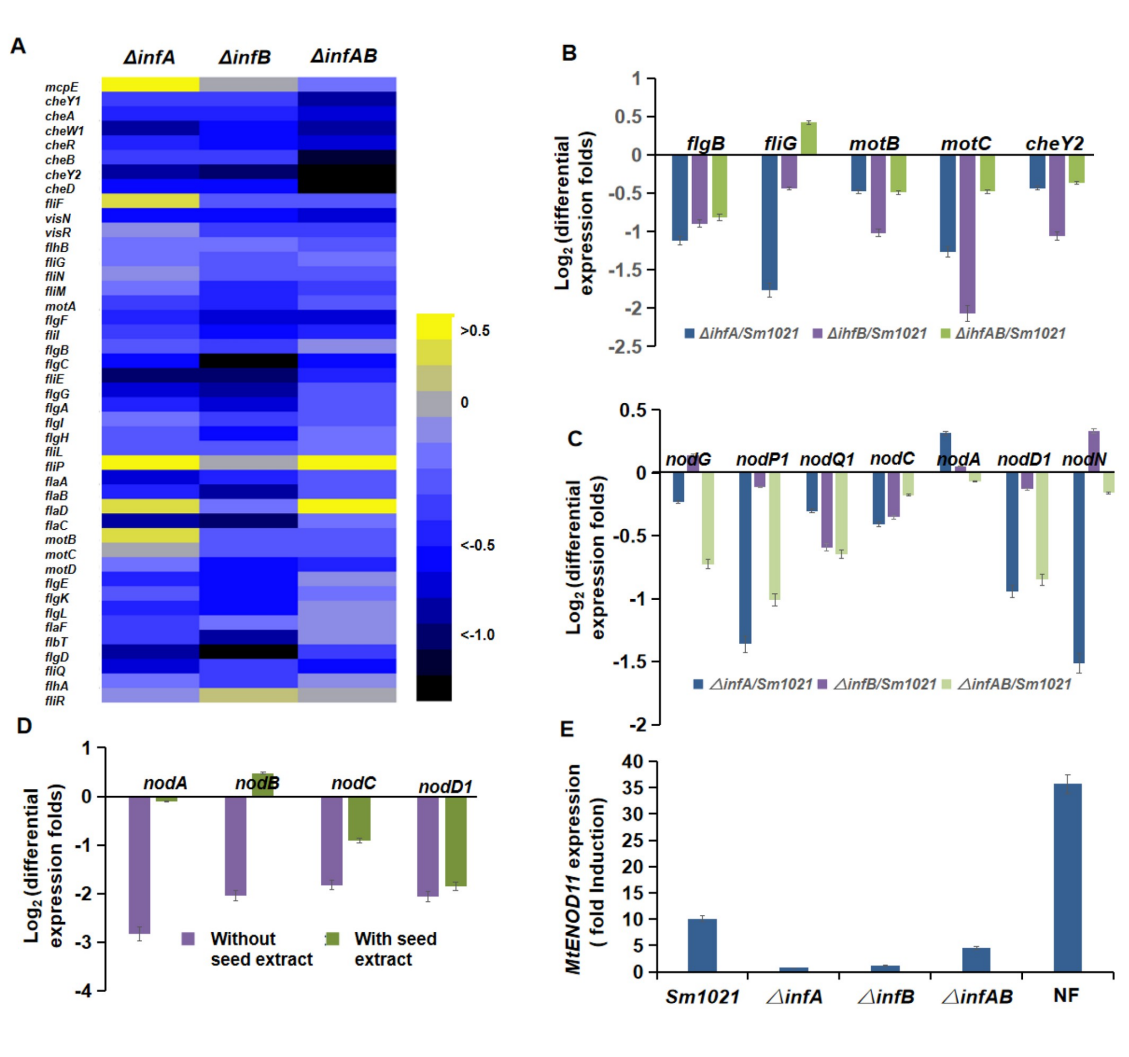
Figure 2 The *ihfA*/ *B* deletion mutants show altered expression of symbiotic genes (A,C) RNA-seq results showing differential expressions of chemotaxis, flagellum and nodulation ( nod) genes in the ihfA/ B mutants relative to the parent strain Sm1021. (B) Expression analysis of five flagellum biosynthesis genes in indicated strains as determined by quantitative RT-PCR. (D) Differential expression of four nodulation genes in the Δ ihfAB mutant versus Sm1021 as analyzed by quantitative RT-PCR. Bacterial cultures were treated with or without alfalfa seed extracts (cells were grown in LB/MC broth to OD 600=0.8). (E) Induction of MtENOD11 expression in M. truncatula after treatment of roots with culture supernatants from indicated S. meliloti strains (cultured in LB/MC broth). For comparison, the Nod factor NodSm-IV (C16:2, S) was applied to the roots at a concentration of 10 –7 M (NF). All RT-PCR experiments were performed in triplicate. The relative expression level for each gene was calculated by using the threshold cycle (2 –ΔΔCt) method. ActinB (for MtENOD11 expression analysis) and the S. meliloti gene SMc00128 (for rhizobial genes) served as the internal reference genes. RT-PCR data are shown as the mean±SD.

As alfalfa nodules induced by *ihfA*/ *B* deletion mutants showed reduced nitrogen fixation ( Figure 1G), we suggest that the lack of *ihf* genes also affects the expressions of nitrogen fixation ( *nif* or *fix*) genes as proposed in a published report [10]. We therefore extracted total RNA from four-week-old alfalfa nodules and performed quantitative RT-PCR for representative *nif* and *fix* genes. The results indicated that *nifA*, *nifD*, *nifH* and *fixT* transcripts were reduced in nodules induced by the *ihfA*/ *B* mutants when compared to nodules harboring Sm1021 ( Supplementary Figure S3). These results are consistent with previous work which reported similar *nif* and *fix* gene expression data in other rhizobial strains [ 5, 6] .

In conclusion, the free-living and symbiotic phenotypes of constructed *S*. *meliloti ihfA*/ *B* deletion mutants showed altered growth and motility properties, reduced the capacity to form effective nitorgen-fixing nodules on alfalfa. Accordingly, expression of *nif* and *fix* genes was reduced in mature nodules. Remarkably, we also found that *ihfA*/ *B* deficient mutants show downregulation of *nod* genes such as *nodD1* and *nodC* which are required for Nod factor production and infection of host legumes. Hence, the *ihfA* and *ihfB* of *S*. *meliloti* can be considered as global transcriptional regulator genes that affect the nodule symbiosis at early and late symbiotic stages.

## Supplementary Data

Supplementary data is available at *Acta Biochimica et Biophysica Sinica* online.
